# Conjunctivitis Granulosa Chronica Treated by Galvanization

**Published:** 1873-01

**Authors:** C. W. Trueheart

**Affiliations:** Surgeon to the “Eye, the Throat and Ear Department” of Galveston City Hospital


### CONJUNCTIVITIS GRANULOSA CHRONICA TREATED BY GALVANIZATION.

BY C. W. TRUEHEART, M.D.,

*Surgeon to the "Eye, the Throat and Ear Department" of Galveston City Hospital.*

It is the object of this paper rather to call attention to the subject considered, and invite experiment at the hands of abler observers possessing better fields for observation, than to make any startling announcement of successes already achieved.

The use of electricity in the treatment of chronic granular conjunctivitis was suggested to me by observing, during recent studies in the wards of the celebrated electro-therapeutist, Professor Dr. Benedick, of Vienna, its wonderful power and efficacy in producing absorption of adventitious formations in other parts of the body. I select the galvanic rather than the faradic current, because with the former, greater molecular activity and "catalytic force" can be excited, and with less pain to the patient, than with the latter.

In carrying out the experiments, I have been careful to select only well-marked cases, and such as had not sustained a loss of tissue by the application of strong caustics. I only regret that the number of suitable cases that have fallen under my care who would submit to the experiments have been so small.

Within the past few months, three cases of this most intractable affection, subjected to the plan of treatment presently to be described, have recovered in about one-fourth the time, and without that destruction of conjunctivial tissue, with its train of evils, attending the usual treatment by caustics.

In sending the current through the upper lid, the one electrode is to be placed just above the middle of the eyebrow; for the lower lid, just over the maxillo-molar articulation; the other electrode, tipped with a piece of delicate sponge, or the hair out of a camel's hair pencil, is to be brushed lightly and slowly over the diseased conjunctiva of the everted lid.

The electrodes should be kept well moistened. I believe it to be a matter of no moment as to the relative positions of the negative and positive poles. The strength of the current used is to be regulated by the patient's sensations of pain, rather than by the galvanometer or the number of elements used.

The application at each sitting should be continued for from ten to thirty minutes, according as the patient can bear it. For the first few sittings, especially where the patient is very sensitive, only a weak current should be used, and the electrodes applied to the integument of the closed lids just over either canthus. Indeed, I think it will, in most cases, be found best to apply the electrodes to the closed lids for the latter part of every sitting, as the application to the everted lids becomes quite painful if continued for more than ten or twelve minutes. Three to five sittings, on consecutive days, will usually serve to excite a sufficiently active inflammatory reaction to set in motion the absorption of diseased materials. A pretty vigorous reaction having been induced, the sitting should be discontinued for three to six or eight days, when, the artificial inflammation having subsided, the sittings are again to be resumed as before. Penciling the conjunctiva daily with a two gr. sol. of arg. nit., in the intermissions of the use of the electricity, seemed to act very well.

The shrinkage of the hypertrophied papillæ and the infiltrated subconjunctival tissue, following each series of sittings, was very marked. In one very severe case of near two years' standing, where the thickening of the lids was such as to insure decided ptosis of both eyes, eight sittings within a period of twenty days caused an improvement of fifty to seventy-five per cent.—*New York Medical Record.*

